# Hoxb5 reprogrammes murine multipotent blood progenitors into haematopoietic stem cell‐like cells

**DOI:** 10.1111/cpr.13235

**Published:** 2022-05-17

**Authors:** Dehao Huang, Qianhao Zhao, Mengyun Zhang, Qitong Weng, Qi Zhang, Kaitao Wang, Fang Dong, Hui Cheng, Fangxiao Hu, Jinyong Wang

**Affiliations:** ^1^ CAS Key Laboratory of Regenerative Biology Guangzhou Institutes of Biomedicine and Health, Chinese Academy of Sciences Guangzhou China; ^2^ University of Chinese Academy of Sciences Beijing China; ^3^ Faculty of Forensic Medicine, Zhongshan School of Medicine Sun Yat‐Sen University China; ^4^ GMU‐GIBH Joint School of Life Sciences Guangzhou Medical University Guangzhou China; ^5^ School of Biomedical Engineering Sun Yat‐Sen University Shenzhen China; ^6^ State Key Laboratory of Experimental Hematology & National Clinical Research Center for Blood Diseases Institute of Hematology & Blood Diseases Hospital, Chinese Academy of Medical Sciences & Peking Union Medical College Tianjin China; ^7^ Center for Stem Cell Medicine & Department of Stem Cell and Regenerative Medicine Chinese Academy of Medical Sciences & Peking Union Medical College Tianjin China; ^8^ State Key Laboratory of Stem Cell and Reproductive Biology, Institute for Stem Cell and Regeneration Institute of Zoology, Chinese Academy of Sciences Beijing China

## Abstract

**Objectives:**

The expression of transcription factor Hoxb5 specifically marks the functional haematopoietic stem cells (HSC) in mice. However, our recent work demonstrated that ectopic expression of Hoxb5 exerted little effect on HSC but could convert B‐cell progenitors into functional T cells *in vivo*. Thus, cell type‐ and development stage‐specific roles of Hoxb5 in haematopoietic hierarchy await more extensive exploration. In this study, we aim to investigate the effect of Hoxb5 expression in multipotent blood progenitor cells.

**Materials and Methods:**

A Mx1cre/Rosa^LSL‐Hoxb5‐EGFP/+^ mouse model was used to evaluate the effect of Hoxb5 expression in blood multipotent progenitor cells (MPP). Golden standard serial transplantation experiments were used to test the long‐term haematopoiesis potential of Hoxb5‐expressing MPP. Single‐cell RNA‐seq analysis was used to characterize the gained molecular features of Hoxb5‐expressing MPP and to compare with the global transcriptome features of natural adult HSC and fetal liver HSC (FL HSC).

**Results:**

Here, with a mouse strain engineered with conditional expression of Hoxb5, we unveiled that induced expression of Hoxb5 in MPP led to the generation of a *de novo* Sca1^+^c‐kit^+^CD11b^+^CD48^+^ (CD11b^+^CD48^+^SK) cell type, which can repopulate long‐term multilineage haematopoiesis in serial transplantations. RNA‐seq analysis showed that CD11b^+^CD48^+^SK cells exhibited acquired features of DNA replication and cell division.

**Conclusions:**

Our current study uncovers that Hoxb5 can empower MPP with self‐renewal ability and indicates an alternative approach for generating HSC‐like cells *in vivo* from blood lineage cells.

## Graphical abstract

We unveiled that induced expression of Hoxb5 in mouse multipotent progenitor cells (MPP) led to the generation of a *de novo* Sca1^+^c‐Kit^+^CD11b^+^CD48^+^ (CD11b^+^CD48^+^SK) cell type, which obtained the self‐renewal ability and can repopulate long‐term multilineage haematopoiesis in serial transplant recipients. RNA‐seq analysis showed that CD11b^+^CD48^+^SK cells exhibited acquired features of DNA replication and cell division, which resembled natural FL HSC. In short, our current study uncovers that Hoxb5 is able to empower MPPs with self‐renewal ability and indicates an alternative approach for generating HSC‐like cells *in vivo* from blood lineage cells.

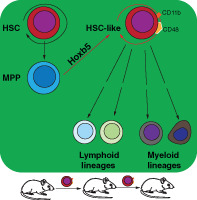

## 1 INTRODUCTION

Haematopoietic stem cell (HSC) is the blood cell type that possesses dual features of self‐renewal and multilineage potential, which are critical for replenishing the entire haematopoietic system throughout an individual lifespan.^1^, ^2^ However, the absolute numbers of HSC in adults are extremely rare^3^, ^4^ and are not efficiently expanded *in vitro*.^5^, ^6^ Researchers have been attempting alternative approaches to generate engraftable blood progenitors by enforcing expressing those molecules highly expressed in HSC but absent in downstream progenies. Ectopic expression of Sox17 can confer self‐renewal potential on adult haematopoietic progenitors. However, this approach eventually led to leukemogenesis.^7^ Likewise, miR‐125a is a non‐coding RNA gene preferentially expressed in HSC rather than blood progenies.^8^ Ectopic expression of miR‐125a in mouse haematopoietic progenitors induced long‐term haematopoiesis, but the recipient mice suffered an MPN‐like disease after secondary transplantation.^9^, ^10^, ^11^ Therefore, more extensive and innovative efforts are needed to develop safer approaches to convert blood progenitor cells into engraftable blood stem cells for ultimately therapeutic uses.

Hoxb5, a member of *HOX* gene family, is preferentially expressed in HSC and uniquely marks the long‐term HSC.^12^, ^13^ Our recent study showed that the gain of function of Hoxb5 in pro‐pre‐B cells reprogrammed these cells into T lymphocytes *in vivo*.^14^ Moreover, the latest research shows that exogeneous Hoxb5 expression confers protection against loss of self‐renewal to Hoxb5‐negative HSCs and can partially alter the cell fate of ST‐HSCs to that of LT‐HSCs.^15^ Here, we further studied the potential role of Hoxb5 in the MPP cell context, an intermediate progeny of HSC without self‐renewal ability. Interestingly, conditional overexpression of Hoxb5 in MPP upon transplantation led to long‐term haematopoiesis in serially transplanted mice. More importantly, Hoxb5 resulted in a *de novo* cell type defined as CD11b^+^CD48^+^SK, which contributed to the sustainable long‐term haematopoiesis in serially transplanted recipients. CD11b^+^CD48^+^SK cells exhibited features related to DNA replication and cell division. This study reveals *de novo* evidence that Hoxb5 can efficiently reprogramme blood progenitors into engraftable blood stem cells.

## 2 MATERIALS AND METHODS

### 2.1 Mice

Animals were housed in the animal facility of the Guangzhou Institutes of Biomedicine and Health (GIBH). Rosa^LSL‐Hoxb5‐EGFP/+^ mice were described as previously reported.^14^ Mice of the CD45.1^+^ and Mx1‐cre strains were purchased from the Jackson laboratory. All the mouse lines were maintained on a pure C57BL/6 genetic background. All experiments were conducted in accordance with experimental protocols approved by the Animal Ethics Committee of GIBH.

### 2.2 Flow cytometry

Antibodies to CD2 (RM2‐5), CD3 (145‐2C11), CD4 (RM4‐5), CD8a (53–6.7), Gr1 (RB6‐8C5), CD11b (M1/70), Ter119 (TER‐119), B220 (6B2), c‐kit (2B8), Sca‐1 (E13‐161.7), CD135 (A2F10), CD150 (TC15‐12F12.2), CD19 (eBio1D3), CD48 (HM48‐1) ki‐67 (16A8), Fcγ RII/III (2.4G2), CD127 (SB/199), CD45.2 (104) and CD45.1(A20) were purchased from eBioscience or BioLegend. DAPI, 7‐AAD or PI was used to stain dead cells. Flow cytometry was performed on an LSR Fortessa (BD Biosciences) and data were processed by FlowJo software (Version: 10.4.0, Tree Star).

### 2.3 Cell sorting

Bone marrow cells used for transplantation or RNA‐seq were first incubated with the biotin‐conjugated antibody to Sca1 (anti‐Sca1 biotin) and then enriched using Anti‐Biotin MicroBeads by autoMACS Pro (Miltenyi Biotec). The enriched cells, stained with antibodies, were sorted by BD FACSAria III.

### 2.4 Transplantation

All recipients (CD45.1^+^, C57BL/6 background) were lethally irradiated (9 Gy, RS2000, Rad Source) at least 4 h, but less than 24 h before transplantation. MPP (400 cells/mouse) from Rosa^LSL‐Hoxb5‐EGFP/+^ mouse or Mx1cre/Rosa^LSL‐Hoxb5‐EGFP/+^ mouse for primary transplantation and donor‐derived CD48^+^CD11b^+^SK cells (2000 cells/mouse) from the primary recipients for secondary transplantation were retro‐orbitally transplanted into the recipients with the enriched Sca1^−^ helper cells (0.25 million/mouse, CD45.1^+^). For third transplantation, total BM cells (10 million/mouse) from the secondary recipients were retro‐orbitally transplanted into the third recipients. To induce Hoxb5 expression, the primary recipients were intraperitoneally injected with polyinosinic‐polycytidylic acid (pIpC) (250 μg/mouse) every other day for six times starting from the 5th day before transplantation, until the 5th day after transplantation. Recipients were fed with the water added with trimethoprim‐sulfamethoxazole for 1 month after irradiation.

### 2.5 RNA‐seq and data analysis

cDNA of the single cell from adult wild‐type HSC (BM HSC, Rosa^LSL‐Hoxb5‐EGFP/+^ mice, 8‐week old), fetal liver HSC (FL HSC, Rosa^LSL‐Hoxb5‐EGFP/+^ mice, Day14.5, defined as CD45.2^+^Lin^−^Sca1^+^c‐kit^+^CD11b^+^CD150^+^), wild‐type MPP (WT‐MPP, Rosa^LSL‐Hoxb5‐EGFP/+^ mice, 8‐week old) and CD48^+^CD11b^+^SK cells (primary recipients, Week 8 post‐transplantation) were extracted and amplified using Discover‐sc WTA Kit V2 (Vazyme). The expression level of B2m and Gapdh was used to assess the quality of the amplified cDNA by qPCR analysis. These qualified samples were used to prepare the sequencing library by the TruePrep DNA Library Prep Kit V2 (Vazyme), and the qualified libraries were sequenced by Illumina sequencer NextSeq 500. Raw data (FASTQ files) were generated using bcl2fastq software (version 2.16.0.10) and were uploaded to the Gene Expression Omnibus public database (GSE 183800). HISAT2 (version 2.1.0) was used to align the raw data, and the StringTie (version 1.3.4) was used to estimate the expression level in the transcripts per million (TPM) as previously reported.^16^, ^17^ Gene set enrichment analysis (GSEA) and gene‐ontology (GO)‐enrichment analysis (clusterProfiler package) were performed as described.^18^, ^19^ Spearman correlation coefficient between population was used for correlation analysis. Correlation analysis was performed by cor() function of R.

## 3 RESULTS

### 3.1 Enforced expression of Hoxb5 in MPP leads to long‐term haematopoiesis in transplantation setting

**FIGURE 1:**
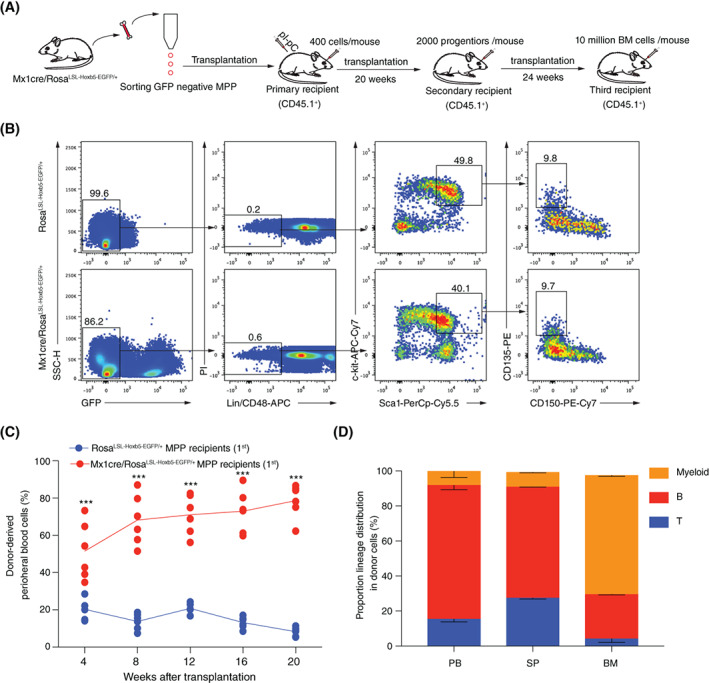
Overexpression of Hoxb5 empowers long‐term reconstitution capacity on MPP. (A) The scheme for MPP transplantation. (B) Gating strategy for sorting the MPP. MPP were defined as CD45.2^+^GFP^−^Lin (CD2^−^CD3^−^CD4^−^CD8^−^CD11b^−^Gr1^−^B220^−^Ter119^−^) CD48^−^Sca1^+^c‐kit^+^CD150^−^CD135^+^ from either the Mx1cre/Rosa^LSL‐Hoxb5‐EGFP/+^ mice or Rosa^LSL‐Hoxb5‐EGFP/+^ control mice (8‐week old). The sorted MPP were transplanted into lethally irradiated (9.0 Gy) recipients (CD45.1^+^, C57BL/6 background, 400 cells/mouse) with Sca1^−^ helper cells (CD45.1^+^, 0.25 million/mouse). Recipients were injected with pIpC (i.p. 250 μg/mouse) every other day for six times starting from day 5 before transplantation. (C) Contribution curves of the donor‐derived cells in peripheral blood cells (PB) of the primary recipients. The donor cells were defined as CD45.2^+^GFP^+^ (Mx1cre/Rosa^LSL‐Hoxb5‐EGFP/+^ MPP recipients) or CD45.2^+^ (Rosa^LSL‐Hoxb5‐EGFP/+^ recipients). The PB of the recipients transplanted with Mx1cre/Rosa^LSL‐Hoxb5‐EGFP/+^ MPP (*n* = 6, as indicated by the red dot) or Rosa^LSL‐Hoxb5‐EGFP/+^ MPP (*n* = 6, as indicated by the blue dot) was analysed every 4 weeks until the Week 20 after transplantation. Mean ± SD, ***p < 0.001 independent samples *t*‐test. (D) Lineage distribution of the recipients (*n* = 3) at Week 20 after Mx1cre/Rosa^LSL‐Hoxb5‐EGFP/+^ MPP transplantation. Columns shown are percentages of donor‐derived T cells (CD3^+^), B cells (CD19^+^) and myeloid cells (CD11b^+^ or Gr1^+^) in PB, spleen (SP) and bone marrow (BM)

To evaluate the potential role of Hoxb5 in MPP, we chose Mx1cre/Rosa^LSL‐Hoxb5‐EGFP/+^ mouse model in which ectopic Hoxb5 was inserted into *Rosa26* in the form of Loxp‐Stop‐Loxp‐Hoxb5‐EGFP cassette as previously reported (Figure S1A).^14^ Thus, the ectopic Hoxb5 can be conditionally induced in the presence of Cre recombinase, which is under the control of the Mx1 promoter, and can be induced by polyinosinic‐polycytidylic acid (pIpC). Upon transplantation into recipient animals, ectopic Hoxb5 expression would be turned on by injection with pIpC, and the GFP signal reports the expression of the ectopic Hoxb5 at single‐cell resolution. We sorted the conventional MPP (CD45.2^+^GFP^−^Lin [CD2, CD3, CD4, CD8, CD11b, Gr1, B220, Ter119]^−^ CD48^−^Sca1^+^c‐kit^+^CD150^−^CD135^+^)^20^, ^21^ from the Sca1^+^ enriched bone marrow cells of Mx1cre/Rosa^LSL‐Hoxb5‐EGFP/+^ mouse or Rosa^LSL‐Hoxb5‐EGFP/+^ mouse. Four hundred sorted MPP along with 0.25 million Sca1^−^ helper cells (CD45.1^+^) were retro‐orbitally transplanted into irradiated individual recipients (CD45.1^+^, C57BL/6 background) (Figure 1A,B). The recipients were intraperitoneally injected with pIpC (250 μg/mouse) every other day for six times starting from day 5 before transplantation. We assessed the reconstitution ability of the donor‐derived cells by analysing the peripheral blood (PB) chimeras every 4 weeks until Week 20 post‐transplantation. (Figure 1A). Amazingly, in the primary recipients transplanted with the MPP of the Mx1cre/Rosa^LSL‐Hoxb5‐EGFP/+^ mouse, the ratio of the donor‐derived cells (CD45.2^+^GFP^+^) continuously increased and the minimum ratio was up to 62% at Week 20 post‐transplantation, whereas the control recipients transplanted with the Rosa^LSL‐Hoxb5‐EGFP/+^ MPP showed a significantly low reconstitution ability, and the maximum donor‐derived cell (CD45.2^+^) ratio was 11.4% at the Week 20 post‐transplantation (Figure 1C). Furthermore, the contributions of donor‐derived cells in the spleen (SP) and bone marrow (BM) tissues of the Hoxb5‐expressing MPP recipients were significantly more than the control group (*p* < 0.001) (Figure S1B). In addition, multiple blood lineages including T cells (CD3^+^), B cells (CD19^+^) and myeloid (CD11b^+^or Gr1^+^) in the PB, SP and BM were also detected at Week 20 post‐transplantation in the primary recipients (Figure 1D). These results demonstrate that enforced expression of Hoxb5 in MPP leads to long‐term haematopoiesis.

### 3.2 Hoxb5 results in the occurrence of a *de novo* CD11b ^+^ CD48 ^+^ SK cell type associated with the long‐term engraftable feature

**FIGURE 2:**
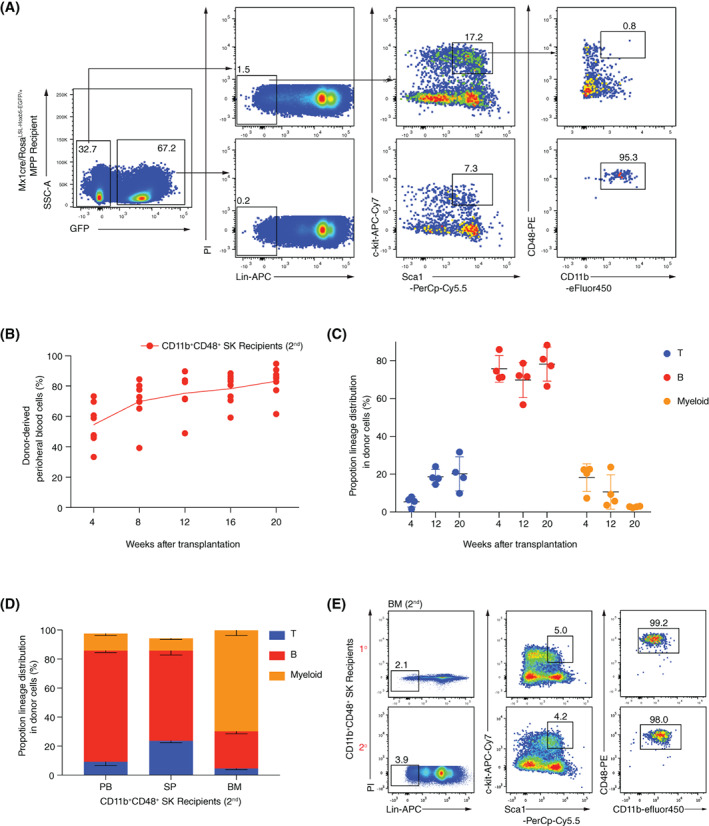
A *de novo* CD11b^+^CD48^+^SK cell population reconstitutes haematopoiesis in secondary recipients. (A) FACS analysis of the donor‐derived BM progenitors in the primary recipients at Week 20 post‐transplantation. Antibodies of lineages (CD2^−^CD3^−^CD4^−^CD8^−^Gr1^−^B220^−^Ter119^−^) (Lin), Sca1, c‐kit, CD11b and CD48 were stained the BM of Mx1cre/Rosa^LSL‐Hoxb5‐EGFP/+^ MPP recipients. CD11b^+^CD48^+^SK cells were defined as CD45.2^+^GFP^+^Lin^−^Sca1^+^c‐kit^+^CD48^+^CD11b^+^ were sorted from the primary recipients at Week 20 post‐transplantation and retro‐orbitally transplanted into the lethally irradiated secondary recipients (CD45.1^+^, C57BL/6 background, 2000 cells/mouse). (B) Chimeras curves of the donor cells to the peripheral blood (PB) cells of the secondary recipients (*n* = 7, as indicated by the red dot). For the secondary transplantation, CD11b^+^CD48^+^SK cells were retro‐orbitally injected into the lethally irradiated recipients (9.0 Gy, 2000 cells/mouse). The donor‐derived cells (CD45.2^+^GFP^+^) in the PB were analysed every 4 weeks post‐transplantation. (C) Lineage distribution in PB of the secondary recipients (*n* = 4) at weeks 4, 12 and 20 post‐transplantation. Proportions of the CD3^+^ (T), CD19^+^ (B) and CD11b^+^ (myeloid) in donor‐derived cells were analysed. Each symbol represents an individual host mouse. (D) Lineage distribution of the recipients (*n* = 3) at Week 24 after CD11b^+^CD48^+^SK cell transplantation. Columns shown are percentages of donor‐derived T cells (CD3^+^), B cells (CD19^+^) and myeloid cells (CD11b^+^ or Gr1^+^) in PB, spleen (SP) and bone marrow (BM). (E) Immunophenotypes of the donor‐derived CD11b^+^CD48^+^SK cells in the bone marrow (BM) of the secondary recipients (two representative mice)

To investigate the cellular mechanism, we analysed the blood progenitor cells in the primary recipients at Week 20 post‐transplantation. We discovered a *de novo* donor‐derived Sca1^+^c‐kit^+^ population cells, which simultaneously expressed CD11b and CD48 surface markers. Certainly, this cell type is not identified in natural blood cells in the absence of Hoxb5 expression (Figure 2A). Consistent with previous reports, natural MPP transplantation cannot sustainably give rise to LSK cells in the bone marrow of recipient mice (Figure S1C). To further test whether the CD11b^+^CD48^+^SK cells are responsible for the long‐term repopulating feature in Hoxb5 expressing MPP, we sorted the GFP^+^CD11b^+^CD48^+^SK cells and transplanted them into secondary recipient mice (CD45.1^+^, C57BL/6 background, 2000 cells/mouse) with Sca1^−^ helper cells (CD45.1^+^, 0.25 million/mouse). As expected, these CD11b^+^CD48^+^SK cells successfully reconstituted multilineage haematopoiesis in secondary recipients, as demonstrated by stable increases of donor‐derived cells (GFP^+^) in the PB after transplantation (Figure 2B). Of note, the donor chimeras achieved as high as 94.6% at Week 20 after transplantation and lineages of T, B and myeloid cells can be detected at Week 4, Week 12 and Week 20 post‐transplantation (Figure 2B,C). Moreover, the donor‐derived T, B and myeloid lineages in the PB, SP and BM also exhibited patterns resembling natural haematopoiesis at the Week 24 post‐transplantation (Figure 2D). More importantly, the donor‐derived CD11b^+^CD48^+^SK cells can still be detected in the BM of the secondary recipients (Figure 2E). These results indicate that the *de novo* CD11b^+^CD48^+^SK cell type is engraftable in the secondary recipients.

**FIGURE 3:**
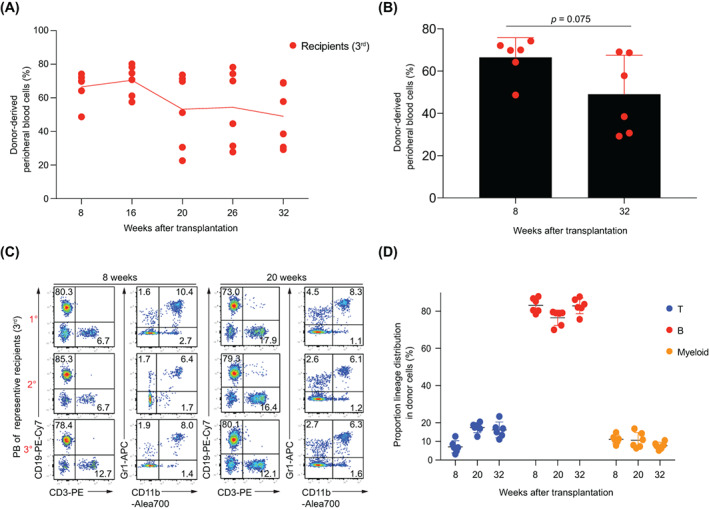
CD11b^+^CD48^+^SK cells still maintained the repopulation capacity in the third transplantation recipients. (A) Chimeric curves of the donor cells in PB of the secondary recipients (*n* = 6, as indicated by the red dot). Donor‐derived cells (CD45.2^+^GFP^+^) in PB were analysed at weeks 8, 16, 20, 26 and 32 post‐transplantation. For the third transplantation, recipients (CD45.1^+^, C57BL/6 background) were lethally irradiated and then were retro‐orbitally injected with the nucleated BM cells (10 million/mouse) isolated from the secondary recipients. (B) Comparison of the donor‐derived cells (CD45.2^+^GFP^+^) at Weeks 8 and 32 post‐transplantation. Mean ± SD, no significant difference, *p* = 0.075, independent samples *t*‐test. (C) Representative FACS analysis (*n* = 3, as indicated by the red dot) of the PB from the third transplantation recipients (3rd) after transplanting with the total BM cells of the secondary recipients at Weeks 8 and 20 post‐transplantation. (D) Lineage distribution in PB of the third recipients (*n* = 6) at Weeks 8, 20 and 32 post‐transplantation. Proportions of CD3^+^ (T), CD19^+^ (B), CD11b^+^or Gr1^+^ (myeloid) in donor‐derived cells were analysed. Each symbol represents an individual host mouse

To assess the long‐term haematopoiesis of the CD11b^+^CD48^+^SK cells, we performed a third round of transplantation using the total BM cells of the CD11b^+^CD48^+^SK cells secondary recipients (10 million/mouse, *n* = 6). The contribution of CD45.2^+^GFP^+^ donor cells to peripheral blood was measured at Weeks 8, 16, 20, 26 and 32 after transplantation. All the recipients were reconstituted with the CD45.2^+^GFP^+^ cells with a ratio range of 48.7%–74.2% at Week 8 post‐transplantation, and the average ratio was still as high as 49% at Week 32 after transplantation (Figure 3A). Moreover, the donor‐derived cell ratio has no significant difference (*p* = .075) at Week 32 compared with Week 8 post‐transplantation (Figure 3B). Meanwhile, the donor‐derived cells also showed multilineage distributions in PB at Weeks 8 and 20 post‐transplantation (Figure 3C). Furthermore, the average ratios of T cells (CD3^+^) were 7.1% (Week 8, *n* = 6), 17.4% (Week 20, *n* = 6) and 16.1% (Week 32, *n* = 6). The average ratios of B cells (CD19^+^) were 83.2% (Week 8, *n* = 6), 76.5% (Week 20, *n* = 6) and 82.8% (Week 32, *n* = 6). As for myeloid cells (CD11b^+^ or Gr1^+^), the average ratios were 11.1% (Week 8, *n* = 6), 10.6% (Week 20, *n* = 6) and 7.7% (Week 32, *n* = 6) post‐transplantation in PB, respectively (Figure 3D). Collectively, these results indicate that the CD11b^+^CD48^+^SK cells can sustain a long‐term haematopoiesis in serial transplantation settings.

### 3.3 Characterization of CD11b ^+^ CD48 ^+^ SK cells at transcriptome level

**FIGURE 4:**
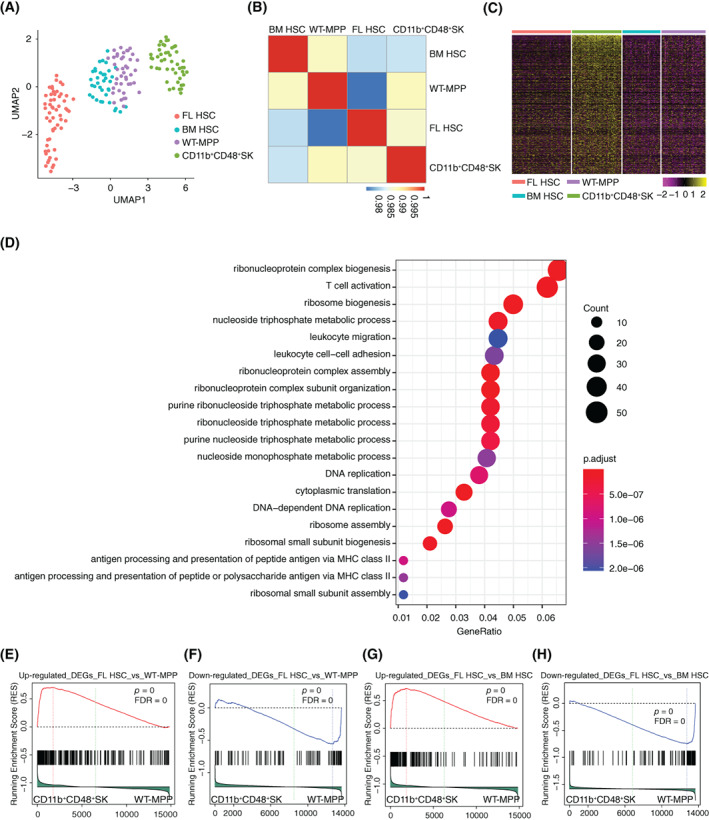
Characterization of CD11b^+^CD48^+^SK cells at single‐cell resolution. (A) UMAP analysis of the WT‐MPP (*n* = 42), BM HSC (*n* = 36), FL HSC (*n* = 56) and CD11b^+^CD48^+^SK cells (*n* = 47). Each colour represents one of the four cell populations. (B) Spearman correlation analysis of the WT‐MPP, BM HSC, FL HSC and CD11b^+^CD48^+^SK cells. (C) Heatmap analysis of CD11b^+^CD48^+^SK cells and other three populations (BM HSC, WT‐MPP and FL HSC). Upregulated differential expressed genes of CD11b^+^CD48^+^SK cells were used for plotting. (D) Gene ontology (GO) enrichment analysis of upregulated DEGs (adjusted *p* value <0.05) in (C) of CD11b^+^CD48^+^SK cells. (E) Gene set enrichment analysis of WT‐MPP (*n* = 42) and CD11b^+^CD48^+^SK cells (*n* = 47). The gene set used for analysis was from the upregulated genes in FL HSC (*n* = 56) versus WT‐MPP (*n* = 42) (adjusted *p* value <0.05). (F) Gene set enrichment analysis of WT‐MPP (*n* = 42) and CD11b^+^CD48^+^SK cells (*n* = 47). The gene set used for analysis was from the downregulated genes in FL HSC (*n* = 56) versus WT‐MPP (*n* = 42) (adjusted *p* value <0.05). (G) Gene set enrichment analysis of WT‐MPP (*n* = 42) and CD11b^+^CD48^+^SK cells (*n* = 47). The gene set used for analysis was from the upregulated genes in FL HSC (*n* = 56) versus BM HSC (*n* = 36) (adjusted *p* value <0.05). (H) Gene set enrichment analysis of WT‐MPP (*n* = 42) and CD11b^+^CD48^+^SK cells (*n* = 47). The gene set used for analysis was from the downregulated genes in FL HSC (*n* = 56) versus BM HSC (*n* = 36) (adjusted *p* value <0.05)

To investigate the underlying molecular mechanisms, we characterized the CD11b^+^CD48^+^SK cells (*n* = 47) at transcriptome levels by single‐cell RNA‐seq analysis. Meanwhile, we also performed single‐cell RNA‐seq of the BM HSC (*n* = 36, Rosa^LSL‐Hoxb5‐EGFP/+^, 8‐week old), WT‐MPP (*n* = 42, Rosa^LSL‐Hoxb5‐EGFP/+^ mice, 8‐week old) and FL HSC (*n* = 56, Rosa^LSL‐Hoxb5‐EGFP/+^ mice, Day 14.5 embryo) (Figure S1D) for comparisons. To unbiasedly cluster the four HSPC populations, UMAP analysis was performed and the results illustrated that CD11b^+^CD48^+^SK cells were artificially unique and distinct from natural FL HSC, BM HSC and WT‐MPP (Figure 4A). The co‐efficiency value between CD11b^+^CD48^+^SK cells and WT‐MPP is 0.988. And the one between CD11b^+^CD48^+^SK cells and FL HSC is 0.987. CD11b^+^CD48^+^SK cells are even closer to WT‐MPP rather than FL HSC (Figure 4B). To further investigate the underlying mechanisms of Hoxb5‐induced CD11b^+^CD48^+^SK cells in long‐term transplantation, we performed differential gene expression analysis of CD11b^+^CD48^+^SK cells with other three populations (FL HSC, BM HSC and WT‐MPP) (Figure 4C), and gene‐ontology (GO) analysis showed that the upregulated genes (adjusted *p* value <0.05) in CD11b^+^CD48^+^SK cells were related to translation‐regulation pathways, T‐cell activation and DNA replication pathways (Figure 4D). To further dissect the transcriptome signatures of CD11b^+^CD48^+^SK cells, we enriched the differentially expressed genes (adjusted *p* value <0.05) between FL HSC and WT‐MPP. The up‐ and downregulated differential expressed genes enriched above were respectively used as gene sets for Gene set enrichment analysis (GSEA) of CD11b^+^CD48^+^SK cells and WT‐MPP (Figure 4E,F). The results showed that the upregulated genes were enriched in the CD11b^+^CD48^+^SK cells (Figure 4E). Meanwhile, we also enriched the up‐ and downregulated differential expressed genes (adjusted *p* value <0.05) between FL HSC and BM HSC for GSEA between CD11b^+^CD48^+^SK cells and BM‐HSC. The GSEA suggested that the upregulated genes in FL HSC were also enriched in the CD11b^+^CD48^+^SK cells (Figure 4G,H). Moreover, we combined the leading edge genes upregulated in FL HSC compared with WT‐MPP or BM HSC and performed heatmap analysis for the four populations. The results showed that the expression levels of these genes in CD11b^+^CD48^+^SK cells were equivalent to those in FL HSC (Figure S1E). We further performed GO analysis using the leading edge genes and observed that they were involved in cell proliferation processes of DNA replication and chromosome segregation (Figure S1F). Besides the higher expression of Hoxb5 in CD11b^+^CD48^+^SK cells and FL HSC when compared with BM HSC and WT‐MPP, several genes regulating the cell cycle and haematopoiesis were also upregulated, including *Birc5*,^22^ *Gmnn*,^23^ *Cdt1*,^23^ *Cdc45* ^24^ and *Gins1* ^25^ (Figure S1G). Furthermore, the essential genes in regulating HSC homeostasis or development, including *Cdk6*,^26^ *Satb1*,^27^, ^28^ *Runx3*,^29^ and *Mybl2* ^30^, ^31^, were exclusively upregulated in CD11b^+^CD48^+^SK cells (Figure S1H). Thus, despite CD11b^+^CD48^+^SK cells demonstrating coefficiency with natural MPP cells, they also share some common features with FL HSC, which might account for their acquired self‐renewal ability.

## 4 DISCUSSION

In this study, we explored the role of Hoxb5 in the MPP cell context. Hoxb5 expression leads to long‐term haematopoiesis of MPP in serial transplantation settings by generating a population of phenotypic CD11b^+^CD48^+^ SK cells. The Hoxb5‐induced CD11b^+^CD48^+^ SK cells shared mixed features of natural MPP and FL HSC, especially the molecular signatures of cell division and proliferation. The stemness feature is the only functional difference between HSC and their progeny MPP. However, the stemness‐losing mechanism along the differentiation path from HSC to MPP is unknown. The genes which are shut down or downregulated in MPP, such as *Hoxb5*, might be accountable for the loss of stemness from HSC to MPP. Moreover, this maybe indicated by the latest research, which reported that exogeneous Hoxb5 expression confers protection against loss of self‐renewal to Hoxb5‐negative HSC and can partially alter the cell fate of ST‐HSC to that of LT‐HSC.^15^ Despite overexpressing Hoxb5 in MPP generated no phenotypic HSC, the *de novo* CD11b^+^CD48^+^SK cells can sustain long‐term engraftment with full blood lineage differentiation potential. Interestingly, FL HSC shared two features with Hoxb5‐expressing CD11b^+^CD48^+^SK cells, demonstrated by rapid proliferation and expressing CD11b surface marker.^32^, ^33^ But BM HSC lose the expression of CD11b,^1^ which is consistent with their predominant dormancy under homeostasis. Therefore, the expression of CD11b is phenotypically associated with the fast‐expanding features of FL HSC and Hoxb5‐expressing CD11b^+^CD48^+^SK cells. Seemingly, the enforced expression of Hoxb5 in MPP activates a cell division machinery^34^, ^35^ without compromising their multilineage differentiation potential. However, the CD11b^+^CD48^+^SK cells have a differentiation bias towards lymphopoiesis, especially the B lymphopoiesis, which was consistent with the results of Hoxb5‐expressing total BM transplantation from the Vavcre/Rosa^LSL‐Hoxb5‐EGFP/+^ as we previously reported,^14^ which also showed no T lineage‐biased feature. Furthermore, there was also no T lineage‐biased differentiation in CD19cre/Rosa^LSL‐Hoxb5‐EGFP/+^mouse, though T lymphocytes were abundantly produced in the recipients which were transplanted with pro‐pre‐B cells sorted from CD19cre/Rosa^LSL‐Hoxb5‐EGFP/+^ mouse. Hence, these results suggested that Hoxb5 empowers different cell types with different cell fates.

Reportedly, ectopic expression of either Sox17 or miR‐125a in MPP can confer a self‐renewal ability but eventually resulted in haematological malignancies. Sox17 eventually led to leukemogenesis within 374 days after transplantation and MiR‐125a‐induced MPN displayed a complex manner of oncogene dependency.^7^, ^36^ Interestingly, no haematological malignancies were found in the recipients transplanted with the Hoxb5‐expressing MPP. Thus, the self‐renewal feature activated by Hoxb5 might be insulated from oncogenesis.

We also tested the engraftment potential of HOXB5‐expressing human MPP in immunodeficient animals. Unfortunately, the HOXB5‐expressing human MPP failed to recapitulate the long‐term engraftment phenotype of Hoxb5‐expressing murine MPP (data not shown). One possible reason is that the function of HOXB5 is not conservative between human and mouse species. However, we cannot exclude another possibility that HOXB5‐overexpressing human MPP need a humanized bone marrow micro‐environment for HOXB5‐reprgramming, which is not available in current immunodeficient animal models.

In conclusion, our study reveals a rare role of Hoxb5 in empowering self‐renewal capacity on MPP, which provides insights into converting blood progenitors into alternative engraftable cell sources.

## AUTHOR CONTRIBUTIONS

Dehao Huang and Qianhao Zhao are co‐first authors. Jinyong Wang, Fangxiao Hu and Qianhao Zhao designed the project. Fangxiao Hu, Qi Zhang and Dehao Huang conducted all the experiments and data analysis. Mengyun Zhang performed the RNA‐seq experiments and Qitong Weng analysed the RNA‐seq data. Qi Zhang performed a part of the mouse genotype experiments. Kaitao Wang and Qitong Weng performed the irradiation experiments. Hui Cheng, Fang Dong, Fangxiao Hu and Jinyong Wang discussed the data. Fangxiao Hu and Jinyong Wang wrote the manuscript and approved it.

## CONFLICT OF INTEREST

The authors declare that there are no competing financial interests in relation to the work described.

## Supporting information


Figure S1

## Data Availability

The Raw data of the RNA‐seq analysis was available in the GEO database (GSE 183800). Other data that support the findings of this study are available within the article or available from the authors upon request.

## References

[1] Morrison SJ , Weissman IL . The long‐term repopulating subset of hematopoietic stem cells is deterministic and isolatable by phenotype. Immunity. 1994;1:661‐673. doi:10.1016/1074-7613(94)90037-x 7541305

[2] Seita J , Weissman IL . Hematopoietic stem cell: self‐renewal versus differentiation. Wiley Interdiscip Rev Syst Biol Med. 2010;2:640‐653. doi:10.1002/wsbm.86 20890962 PMC2950323

[3] Abkowitz JL , Catlin SN , McCallie MT , Guttorp P . Evidence that the number of hematopoietic stem cells per animal is conserved in mammals. Blood. 2002;100:2665‐2667. doi:10.1182/blood-2002-03-0822 12239184

[4] Bernitz JM , Kim HS , MacArthur B , Sieburg H , Moore K . Hematopoietic stem cells count and remember self‐renewal divisions. Cell. 2016;167:1296, e1210‐1309. doi:10.1016/j.cell.2016.10.022 27839867 PMC5115957

[5] Tajer P , Pike‐Overzet K , Arias S , Havenga M , Staal FJT . Ex vivo expansion of hematopoietic stem cells for therapeutic purposes: lessons from development and the niche. Cell. 2019;8(2):169. doi:10.3390/cells8020169 PMC6407064 30781676

[6] Kumar S , Geiger H . HSC niche biology and HSC expansion ex vivo. Trends Mol Med. 2017;23:799‐819. doi:10.1016/j.molmed.2017.07.003 28801069 PMC5600322

[7] He S , Kim I , Lim MS , Morrison SJ . Sox17 expression confers self‐renewal potential and fetal stem cell characteristics upon adult hematopoietic progenitors. Genes Dev. 2011;25:1613‐1627. doi:10.1101/gad.2052911 21828271 PMC3182027

[8] Guo S , Lu J , Schlanger R , et al. MicroRNA miR‐125a controls hematopoietic stem cell number. Proc Natl Acad Sci USA. 2010;107:14229‐14234. doi:10.1073/pnas.0913574107 20616003 PMC2922532

[9] Gerrits A , Walasek MA , Olthof S , et al. Genetic screen identifies microRNA cluster 99b/let‐7e/125a as a regulator of primitive hematopoietic cells. Blood. 2012;119:377‐387. doi:10.1182/blood-2011-01-331686 22123844

[10] Wojtowicz EE , Walasek MA , Broekhuis MJC , et al. MicroRNA‐125 family members exert a similar role in the regulation of murine hematopoiesis. Exp Hematol. 2014;42:909, e901‐918. doi:10.1016/j.exphem.2014.06.010 25092555

[11] Wojtowicz EE , Lechman ER , Hermans KG , et al. Ectopic miR‐125a expression induces long‐term repopulating stem cell capacity in mouse and human hematopoietic progenitors. Cell Stem Cell. 2016;19:383‐396. doi:10.1016/j.stem.2016.06.008 27424784 PMC5500905

[12] Chen JY , Miyanishi M , Wang SK , et al. Hoxb5 marks long‐term haematopoietic stem cells and reveals a homogenous perivascular niche. Nature. 2016;530:223‐227. doi:10.1038/nature16943 26863982 PMC4854608

[13] Gulati GS , Zukowska M , Noh JJ , et al. Neogenin‐1 distinguishes between myeloid‐biased and balanced Hoxb5 (+) mouse long‐term hematopoietic stem cells. Proc Natl Acad Sci USA. 2019;116:25115‐25125. doi:10.1073/pnas.1911024116 31754028 PMC6911217

[14] Zhang M , Dong Y , Hu F , et al. Transcription factor Hoxb5 reprograms B cells into functional T lymphocytes. Nat Immunol. 2018;19:279‐290. doi:10.1038/s41590-018-0046-x 29434353 PMC6190911

[15] Sakamaki T , Kao KS , Nishi K , et al. Hoxb5 defines the heterogeneity of self‐renewal capacity in the hematopoietic stem cell compartment. Biochem Biophys Res Commun. 2021;539:34‐41. doi:10.1016/j.bbrc.2020.12.077 33418191

[16] Pertea M , Pertea GM , Antonescu CM , Chang TC , Mendell JT , Salzberg SL . StringTie enables improved reconstruction of a transcriptome from RNA‐seq reads. Nat Biotechnol. 2015;33:290‐295. doi:10.1038/nbt.3122 25690850 PMC4643835

[17] Pertea M , Kim D , Pertea GM , Leek JT , Salzberg SL . Transcript‐level expression analysis of RNA‐seq experiments with HISAT, StringTie and Ballgown. Nat Protoc. 2016;11:1650‐1667. doi:10.1038/nprot.2016.095 27560171 PMC5032908

[18] Subramanian A , Tamayo P , Mootha VK , et al. Gene set enrichment analysis: a knowledge‐based approach for interpreting genome‐wide expression profiles. Proc Natl Acad Sci USA. 2005;102:15545‐15550. doi:10.1073/pnas.0506580102 16199517 PMC1239896

[19] Yu G , Wang LG , Han Y , He QY . clusterProfiler: an R package for comparing biological themes among gene clusters. Omics. 2012;16:284‐287. doi:10.1089/omi.2011.0118 22455463 PMC3339379

[20] Kiel MJ , Yilmaz ÖH , Iwashita T , Yilmaz OH , Terhorst C , Morrison SJ . SLAM family receptors distinguish hematopoietic stem and progenitor cells and reveal endothelial niches for stem cells. Cell. 2005;121:1109‐1121. doi:10.1016/j.cell.2005.05.026 15989959

[21] Adolfsson J , Borge OJ , Bryder D , et al. Upregulation of Flt3 expression within the bone marrow Lin(−)Sca1(+)c‐kit(+) stem cell compartment is accompanied by loss of self‐renewal capacity. Immunity. 2001;15:659‐669. doi:10.1016/s1074-7613(01)00220-5 11672547

[22] Gurbuxani S , Xu Y , Keerthivasan G , Wickrema A , Crispino JD . Differential requirements for survivin in hematopoietic cell development. Proc Natl Acad Sci USA. 2005;102:11480‐11485. doi:10.1073/pnas.0500303102 16055565 PMC1183538

[23] Yasunaga S , Ohno Y , Shirasu N , et al. Role of geminin in cell fate determination of hematopoietic stem cells (HSCs). Int J Hematol. 2016;104:324‐329. doi:10.1007/s12185-016-2060-9 27422432

[24] Flach J , Bakker ST , Mohrin M , et al. Replication stress is a potent driver of functional decline in ageing haematopoietic stem cells. Nature. 2014;512:198‐202. doi:10.1038/nature13619 25079315 PMC4456040

[25] Ueno M , Itoh M , Sugihara K , Asano M , Takakura N . Both alleles of PSF1 are required for maintenance of pool size of immature hematopoietic cells and acute bone marrow regeneration. Blood. 2009;113:555‐562. doi:10.1182/blood-2008-01-136879 18984863

[26] Scheicher R , Hoelbl‐Kovacic A , Bellutti F , et al. CDK6 as a key regulator of hematopoietic and leukemic stem cell activation. Blood. 2015;125:90‐101. doi:10.1182/blood-2014-06-584417 25342715 PMC4281832

[27] Yasui D , Miyano M , Cai S , Varga‐Weisz P , Kohwi‐Shigematsu T . SATB1 targets chromatin remodelling to regulate genes over long distances. Nature. 2002;419:641‐645. doi:10.1038/nature01084 12374985

[28] Will B , Vogler TO , Bartholdy B , et al. Satb1 regulates the self‐renewal of hematopoietic stem cells by promoting quiescence and repressing differentiation commitment. Nat Immunol. 2013;14:437‐445. doi:10.1038/ni.2572 23563689 PMC3633104

[29] de Bruijn M , Dzierzak E . Runx transcription factors in the development and function of the definitive hematopoietic system. Blood. 2017;129:2061‐2069. doi:10.1182/blood-2016-12-689109 28179276

[30] Baker SJ , Ma'ayan A , Lieu YK , et al. B‐myb is an essential regulator of hematopoietic stem cell and myeloid progenitor cell development. Proc Natl Acad Sci USA. 2014;111:3122‐3127. doi:10.1073/pnas.1315464111 24516162 PMC3939923

[31] Bayley R , Blakemore D , Cancian L , et al. MYBL2 supports DNA double Strand break repair in hematopoietic stem cells. Cancer Res. 2018;78:5767‐5779. doi:10.1158/0008-5472.CAN-18-0273 30082276

[32] Morrison SJ , Hemmati HD , Wandycz AM , et al. The purification and characterization of fetal liver hematopoietic stem cells. Proc Natl Acad Sci USA. 1995;92:10302‐10306. doi:10.1073/pnas.92.22.10302 7479772 PMC40784

[33] Kim I , He S , Yilmaz OH , Kiel MJ , Morrison SJ . Enhanced purification of fetal liver hematopoietic stem cells using SLAM family receptors. Blood. 2006;108:737‐744. doi:10.1182/blood-2005-10-4135 16569764 PMC1895480

[34] Dalton S . Linking the cell cycle to cell fate decisions. Trends Cell Biol. 2015;25:592‐600. doi:10.1016/j.tcb.2015.07.007 26410405 PMC4584407

[35] Gao SW , Liu F . Novel insights into cell cycle regulation of cell fate determination. J Zhejiang Univ Sci B. 2019;20:467‐475. doi:10.1631/jzus.B1900197 31090272 PMC6568219

[36] Guo S , Bai H , Megyola CM , et al. Complex oncogene dependence in microRNA‐125a‐induced myeloproliferative neoplasms. Proc Natl Acad Sci U S A. 2012;109:16636‐16641. doi:10.1073/pnas.1213196109 23012470 PMC3478612

